# NMDA antagonist agents for the treatment of symptoms in autism spectrum disorder: a systematic review and meta-analysis

**DOI:** 10.3389/fphar.2024.1395867

**Published:** 2024-07-23

**Authors:** Marie-Lou Dessus-Gilbert, Mikail Nourredine, Luc Zimmer, Benjamin Rolland, Marie-Maude Geoffray, Marine Auffret, Lucie Jurek

**Affiliations:** ^1^ Faculté de médecine, Université Claude Bernard Lyon 1, Lyon, France; ^2^ Hospices Civils de Lyon, Lyon, France; ^3^ Laboratoire d’évaluation et modélisation des effets thérapeutiques, UMR CNRS, Lyon, France; ^4^ Lyon Neurosciences Research Center (CRNL), INSERM, CNRS, Lyon, France; ^5^ Centre Hospitalier Le Vinatier, Bron, France; ^6^ RESHAPE U1290, Université Claude Bernard Lyon 1, Lyon, France

**Keywords:** autism, d-cycloserine, amantadine, memantine, meta-analysis, NMDA

## Abstract

**Aims:**

This systematic review and meta-analysis aimed to assess the efficacy of NMDA antagonists in ASD (Autism Spectrum Disorder) on the core (communication and social interaction, repetitive behavior) and associated symptoms (irritability) of ASD, as well as their safety.

**Methods:**

PubMed, CENTRAL, CINHAL, EMBASE, and PsycINFO databases were searched until November 2023. Two authors independently selected the studies and extracted data. Randomized controlled trials assessing the efficacy of NMDA receptor antagonists in participants with ASD aged <18 years were included. The quality of the studies was assessed using the Risk of Bias-2 tool. A random-effect meta-analysis model was used to calculate standardized mean differences (SMD) or odds ratios (OR) using meta package in R.

**Results:**

This systematic review included ten studies (588 participants). Most studies did not report scales assessing core symptoms of ASD. Meta-analysis of efficacy on ASD core symptoms included three studies (248 participants). NMDA antagonists were not superior to placebo [SMD = 0.29; CI 95% (−1,94; 1.35); I^2^ = 0%]. NMDA antagonists was not superior to placebo concerning response (four studies, 189 participants) [OR = 2.4; CI 95% (0.69; 8.38); I^2^ = 35%]. Meta-analysis of efficacy on irritability included three studies (186 participants); NMDA antagonists were not superior to placebo [MD irritability = −1.94; CI 95% (−4.66; 0.77); I^2^ = 0%]. Compared with placebo, significantly more participants in the NMDA antagonist group reported at least one adverse event (five studies, 310 participants) [OR = 2.04; CI 95% (1.17; 3.57); I^2^ = 0%].

**Conclusion:**

Current evidence does not support the effectiveness of NMDA antagonists in the treatment of ASD symptoms or irritability. Further research is needed due to the limited and low quality data available.

**Systematic Review Registration:**

PROSPERO CRD42018110399.

## Introduction

Autism Spectrum Disorder (ASD) is a neurodevelopmental disorder characterized by communication and social interaction deficits, as well as repetitive behaviors and/or restricted interests, referred to as core symptoms of ASD (American Psychiatric Association and éditeur, 2015). The proportion of autistic children in the general pediatric population is estimated to be 0.6% in Europe and 0.4% worldwide (Salari et al., 2022).

While only two medications are approved by the FDA (risperidone and aripiprazole) for the treatment of irritability associated with ASD, no pharmacological treatment is indicated for the decrease in ASD core symptoms (Center for Drug Evaluation and Research and U.S. Food and Drug Administration, 2018).

Studies of neurotransmitters and autism have suggested that aberrant glutamatergic transmission may play a role in ASD. Glutamate is an important excitatory neurotransmitter essential to cognitive function and neuronal development. Its action on neuroglial cells has various effects, such as neuronal migration, differentiation, and development. The glutamatergic system contributes to neural plasticity and cognitive functions. However, excess glutamate can be neurotoxic, leading to cellular death (Rojas, 2014; Uzunova et al., 2014).

Several experimental studies have found abnormalities in the glutamate system in ASD, mainly focusing on the NMDA (N-methyl-D-aspartate) receptor, an ionotropic receptor that enhances glutamatergic excitation. *Postmortem* studies of the brain tissues of autistic patients, for example, have shown lower levels of glutamate decarboxylase, the catalyst that converts glutamate to GABA, and increased NMDA receptor density (Purcell et al., 2001; Rojas, 2014). Dysfunction in NMDA receptors at excitatory synapses has been associated with ASD (Ghanizadeh, 2011; Burnashev and Szepetowski, 2015). Genetic studies have shown alterations in NMDA receptor subunit in ASD (Paoletti et al., 2013). Animal models of ASD suggest bidirectional dysfunction of NMDA receptors by showing, among other things, that modulators of NMDA receptors can normalize ASD-like behaviors in animal models (Kang and Kim, 2015; Lee et al., 2015; Chung et al., 2019).

Different NMDA antagonist drugs that act on the glutamatergic system by blocking glutamate entry into cells have been assessed in ASD. This review aims to appraise the efficacy and safety of NMDA antagonists on ASD symptom severity in autistic children. Efficacy on behavioral problem outcomes (irritability/hyperactivity) will also be evaluated.

## Methods

### Protocol and registration

The recommendations of the Preferred Reporting Items for Systematic Reviews and Meta-analyses (PRISMA 2020) (Page et al., 2021) reporting guideline were followed herein (see Supplementary Material). The protocol of this review was registered in PROSPERO in October 2018 (CRD42018110399). Deviations from the preregistered protocol are described below.

### Search strategy

We have searched the following electronic bibliographic databases: PubMed, the Cochrane Central Register of Controlled Trials (CENTRAL), CINHAL, EMBASE, and PsycINFO, from their creation until October 2, 2020.

A combination of terms related to ASD and NMDA antagonists were used. The complete algorithm is presented in the Supplementary Material. The reference lists of the included articles were manually checked to identify any additional relevant studies.

Alerts were used to retrieve new eligible articles up to November 2023. No new studies were included.

### Study selection

Two independent reviewers (L.J. and M.D.) conducted the literature search with the help of the Covidence website (www.covidence.com) to process the double-blind selection and to manage the duplicates.

Each reviewer checked the relevance of the different studies through their titles and abstracts. The full texts were read to determine their eligibility. Disagreements were resolved by referral to a third author (M.N).

The inclusion criteria were as follows: 1) original articles written in English or French, published in a peer-reviewed journal reporting randomized controlled studies (RCT) or unpublished trials retrieved from CENTRAL if results were available; 2) the population was composed of children (under 18 years of age) with a clinical diagnosis of ASD (or Pervasive Developmental Disorders, PDD) corresponding to the criteria of the Diagnostic and Statistical Manual for Mental Disorders (DSM-IV), Fourth or Fifth (DSM-5) Editions, or the International Classification of Disease, 10 th Revision (ICD-10), or using a standardized diagnostic instrument. Genetic syndromes and ADHD were accepted if associated with a documented diagnosis of ASD or PDD; 3) The intervention was a pharmacological intervention with an NMDA antagonist (e.g., memantine, dextromethorphan, atomoxetine, ketamine, amantadine, acamprosate, felbamate, minocycline, d-cycloserine, lanicomine, nitrous oxide, taxoprodil, or rapastinel). Any dosage, duration, or administration frequency of the drug was considered. The control procedure was a placebo.

Animal studies, studies including adults and the elderly, or studies on autistic symptoms without ASD (or PDD) were excluded from this review.

### Data extraction

For each included study, two reviewers (L.J. and ML.D.) extracted the following variables using a standardized extraction form: study design, sample size, population characteristics, ASD diagnosis method, adverse events, and study results.

The extracted data were verified by a third author (M. N.). In case of missing data or additional details, the primary authors were contacted by mail or directly by telephone. Eight authors were contacted, but no responses were received.

### Bias and quality assessment

Two independent reviewers (L. J. and M. N.) assessed the risk of bias of the included studies using the Revised Cochrane risk-of-bias tool for randomized trials (RoB 2). Any discrepancies were resolved through discussion with a third author (M.G.). The ROB2 contains five domains, as follows: 1) Bias arising from the randomization process, 2) Bias due to deviations from intended interventions, 3) Bias due to missing outcome data, 4) Bias in measurement of the outcome, 5) Bias in selection of the reported result. Each domain is divided into signage questions. The response options for the signaling questions are: 1) Yes, 2) Probably yes, 3) Probably no, 4) No, and 5) No information. These responses allow us to determine a Low, Unclear, or High risk of bias.

### Statistical analysis

Data were analyzed using R studio (R software version 4.1.2) with the “meta” package (version 6.5–0). We used random-effects models because they allow the true population effect size to differ among studies. The effect size was the odds ratio for dichotomous outcomes and mean differences for continuous outcomes. The standardized mean difference was used when all studies assessed the same outcome but used different scales. Restricted maximum likelihood estimator for tau^2^ was used. The Hartung Knapp method (IntHout et al., 2014) was used to compute confidence intervals of the summary effect. Heterogeneity was analyzed using tau^2^ and I^2^.

### Deviation from protocol

The initially planned meta-analysis could not be carried out with all the included studies due to the clinical heterogeneity of the available data. Therefore, we focused on four criteria: efficacy of NMDA antagonists on autism core symptoms, number of responders, efficacy on irritability, and the number of adverse events.

Although initially planned, none of the subgroup or sensitivity analyses were performed because of the small number of available studies (Higgins and Thompson, 2004). Similarly, as previously recommended, publication bias was not assessed because less than ten studies were included in the meta-analysis (Lau et al., 2006).

## Results

### Search results

**FIGURE 1:**
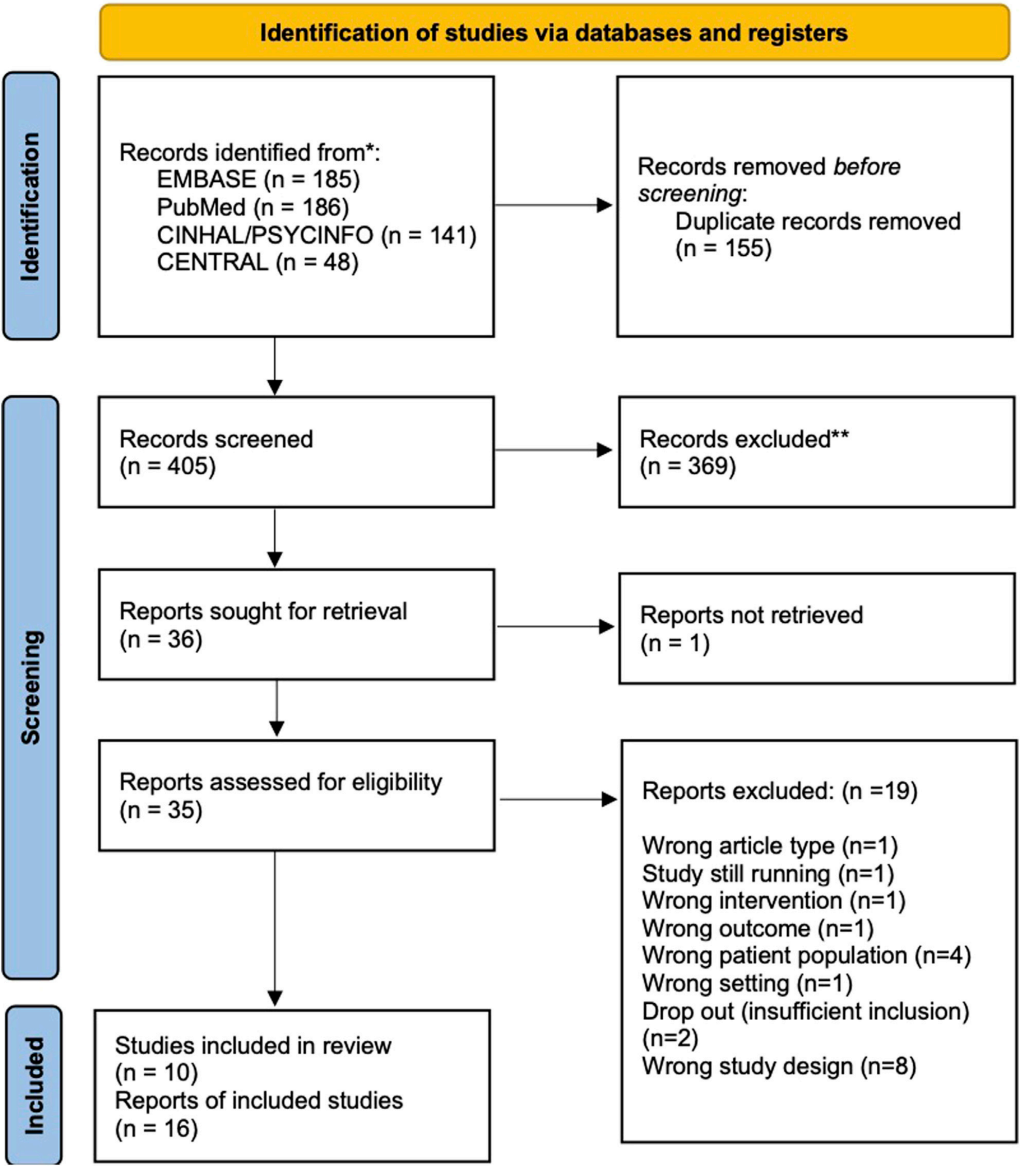
PRISMA flowchart of the selected studies.

The literature search generated 560 articles. Thirteen additional records were identified through manual searches of bibliographies. After removing duplicates, the titles and abstracts of 405 records were screened, and 35 records were assessed for eligibility by full-text review. Nineteen articles were excluded as they did not meet the inclusion criteria. The reasons for exclusion are outlined in Figure 1 and the Supplementary Material. An additional search performed before the final analysis did not retrieve any additional articles. Two publications reported the same study (Minshawi et al., 2016; Wink et al., 2017). The main publication of the RCT (Minshawi et al., 2016) was included; however, information from both publications was used. Four reports, each corresponding to one peer-reviewed publication, were retrieved from the trial registers. Ten unique studies were included in the systematic review, with a total of 588 participants (314 with intervention and 256 with placebo).

### Characteristics of studies included in the systematic review

The characteristics and main results of the included studies are summarized in Table 1.

**TABLE 1:** Characteristic of included studies.

Authors country	Study design (Follow up)	Drug tested, dose and control	Population characteristics	IQ	ADHD	Primary outcome	Global measure	Social interaction	Restricted behavior	Behavioral difficulties	Serious adverse events	ROB
King 2001 United Kingdom	RCT (5W with 1 week placebo lead-in)	Amantadine 2.5–5 mg/kg/d vs. Placebo	N = 39Male = 87.2% Mean age (yo) = 7 (range 5–15) in intervention and 7 (range 5–11) in placebo	Mental age equivalent>18 months or >35 (VABS−2)	Unknown but Psychostimulant exclued	Responder defined by a 25% decrease in parent-rated ABC-C hyperactivity and/or irritabilityAmantadine: 47% Placebo: 37% OR = 1.5 [95% CI (0.4; 5.9)]	CGI-I marked or moderate improvement 53% after amantadine hydrochloride compared with 25% for placebo (*p* = 0.076)	ABC-lethargy: repeated measure (*p* = 0.960) Parent-rated ABC-lethargy: repeated measure (*p* = 0.353)	ABC-Stereotypy: MD = −2.20 (95% CI: 4.74 to 0.33)	ABC hyperactivity MD: 5.75 (95% CI: 11.39 to −0.10) Parent-rated ABC-Hyperactivity MD = −4.81 (95%CI: 11.63 to 2.00) ABC-Irritability: repeated measure (*p* = 0.141)Parent-rated ABC-irritability: repeated measure (*p* = 0.178)	None	High risk
Woodard 2007 United States	RCT ABAB design (4W)	Dextromethorphan: 30–60 mg/d vs. Placebo	N = 16Male = 87.5% Age (yo) = 9–17	Mental age equivalent of 6 months to 4 yo	Unknown	Main outcome not stated	ABC-total and CGI-S (NS)	ABC-Social withdrawal: intervention group mean = 6.59 (SD = 5.02), placebo group mean = 6.91 (SD = 5.63)	ABC-Stereotypy: intervention group mean = 6.22 (SD = 3.78), placebo group mean = 5.78 (SD = 2.73)	ABC-Irritability: intervention group mean = 11.38 (SD = 7.84), placebo group mean = 12.09 (SD = 5.96) ABC-Hyperactivity: intervention group mean = 14.19 (SD = 6.82), placebo group mean = 16.25 (SD = 8.0)	None	High risk
Ghaleiha 2012 Iran Irct1138901151556 N 2010	RCT (10W)	Memantine 5–20 mg/d + Risperidone 3 mg/d vs. Placebo + Risperidone 3 mg/d	N = 40Male = 57.5 Mean age (yo) = 7.42 (SD 1,48) intervention, 7.97 (SD 1,68) placebo	>35	Unknown	ABC-C irritability: repeated measures (*p* ≤ 0.01) Week 10: Placebo + risperidone mean: 12.75 (SD 3.05) Memantine + risperidone mean: 8.90 (SD 1.55)	not assessed	ABC-C social withdrawal: repeated measures (*p* = 0.10) Week 10: Placebo + risperidone mean: 13.85 (SD 2.1) Memantine + risperidone mean: 11.65 (SD 3.39)	ABC-C stereotypy: repeated measures (*p* ≤ 0.01) Week 10: Placebo + risperidone mean: 6.99 (SD 1.97) Memantine + risperidone mean: 3.3 (SD 1.3)	ABC-C irritability: see primary outcome ABC-C hyperactivity: repeated measures (*p* ≤ 0.01) Week 10: Placebo + risperidone mean: 13.85 (SD 3.28) Memantine + risperidone mean: 8.25 (SD 2.19)	None	High risk
Mohammadi 2013 Iran Irct201106101556 N 2011	RCT (10 W)	Amantadine 100–150 mg/d + Risperidone 0.5 – 2 mg/d vs. Risperidone 0.5 – 2 mg/d	N = 40Male = 82.5% Mean age = 6.4 (SD 2,3) intervention, 7.1 (SD 2,4) control	≥35	Exclued	ABC-C Irritability Week 10 (reduction from baseline) Risperidone + Amantadine: 8.60 (SD 4.65) Risperidone + placebo: 5.35 (SD 3.95) MD = 3.2 95% CI 0.48 to 6.01	50% responder in intervention group (defined by a CGI-I much improved or very much improved) and 20% in placebo group [χ2(1) = 3.956, *p* = 0.047]	ABC-C Social withdrawalWeek 10 (reduction from baseline) Risperidone + Amantadine: 1.35 (SD 3.18) Risperidone + placebo: 1.30 (SD 3.33) MD = 0.05 (CI 95% = −2.03–2.13)	ABC-C StereotypyWeek 10 (reduction from baseline) Risperidone + Amantadine: 1.20 (SD 2.33) Risperidone + placebo: 1.20 (SD 2.09) MD = 0.00 (CI 95% = −1.41 to 1.41)	ABC-C irritability: see primary outcome ABC-C Hyperactivity Week 10 (reduction from baseline) Risperidone + Amantadine: 6.15 (SD 5.11) Risperidone + placebo: 2.50 (SD 5.0) MD: 3.65 (CI 95% = 0.41–6.88)	None	Low risk
Aman 2015 United States NCT00872898	RCT (12W)	Memantine 3–15 mg/d vs. placebo	N = 121 Male = 83.5% Mean age = 9.0 (SD 2,2) intervention, 8.9 (SD 2,2) placebo	Mean 77.9 ± 23.1 intervention, 75.7 ± 19.4 placebo	Unknown (4 methylphenidate use)	SRS week 12: MD = −0.1 [−7.2, 6.6]	SRS total score see primary outcome CATS-I total score week 12: MD = −2.2 (95% CI = −4.9 to 0.6) CGI-I not reported	CATS-I social interaction week 12: MD = −1.4 (95% CI = −3.2 to 0.5) CCC initiation week 12: MD = −0.0 (95% CI = −1.3 to 1.3) ABC not reported	CCC interests week 12:MD = −0.0 (95% CI = −1.2 to 1.1) ABC not reported	ABC not reported	2 serious adverse events (Affective disorder and lobar pneumonia) with Memantine	Moderate risk
Martsenkovsky 2016 Ukraine	RCT (16 W)	Memantine 3 – 15 mg/d + ABA (10–20 h/w) vs. ABA (10–20 h/w)	N = NAMale = NAAge = 18–36 months	Unknown	Unknown	Main outcome not stated	not reported	ABC-lethargy/social withdrawal (F = 2.44, df. = 1.52, *p* = 0.10)	ABC-stereotypic behavior (F = 27.11, df. = 1.47, *p* < 0.01)	ABC-irritability (F = 20.34, df. = 1.73, *p* < 0.001) ABC-hyperactivity (F = 143.30, df. = 1.61, *p* < 0.01)	No serious adverse events	High risk
Minshawi 2016 Wink 2017 United States	RCT (10W)	D-Cycloserine 50 mg/d + social skills training vs. social skills training + placebo	N = 67 Male = 82.1% Mean age = 8.38 (SD 1.93) intervention, 8.25 (SD 1.73) placebo	>70 – mean 92.42 (17.76) intervention, 87.30 (15.74) placebo	Unknown	SRS parent-rated at week 11 MD 3.61 95%CI [−5.95 to 13.17]	SRS total score see primary outcome VABS total score at week 11 MD = −0.83 (95%CI = −20.66 to 19.00)	ABC-social withdrawal at week 11MD = −0.68 (95%CI = −3.71 to 2.34)	ABC-stereotypy at week 11 MD = 0.12 (−1.81–2.04)	ABC-irritability at week 11MD = −0.32 (−3.37 to 2.73) ABC-hyperactivity at week 11MD = 0.49 (95% CI −3.72–4.69)	Irritability	Moderate risk
Karahmadi 2018Iran	RCT (12W)	Memantine 5–20 mg/d + ABA vs. ABA+ placebo	N = 60Male = 76.7%Mean age (yo) = 10 (SD 3.48) intervention, 9.5 (SD 3,86) placebo	Unknown	Excluded	Main outcome not stated	GARS total score at 12 weeks: intervention group 73.5 ± 9.81placebo group 89.63 ± 13.95*p* < 0.001	GARS social interactions at 12 weeks: intervention group 23.67 ± 2.66placebo group 31.20 ± 8.02*p* < 0.001	GARS stereotyped behaviors at 12 weeks: intervention group 18.90 ± 3.68placebo group 27.37 ±8.58*p* < 0.001	not assessed	No serious adverse events	High risk
Gagan 2019 United States NCT01972074	RCT (12W)	Memantine, max 20 mg/d vs. placebo	N = 43Male = 77.3%Mean age (yo) = 13.2 (SD 2.7) intervention, 13.3 (SD 2.5) placebo	>70	Unknown	Main outcome: treatment responder (25% reduction on SRS and CGI-I ≤2): 47% with Memantine vs. 19% with Placebo	not reported	not reported	not reported	not reported	No serious adverse events	High risk
Hardan 2019 United States NCT01592747	RCT Withdrawal study from responder of a previous RCT (12W)	Full dose memanine vs. Memantinereduced-dose vs. placebo	N = 144Male = 85.1%Age range (yo) = 6–12 yo	>50, Mean = 91.1 (25.4)	Unknown	Loss of therapeutic response >9-point increase in SRS total raw score at any visit during 12 weeks: Placebo: 69 %Full dose: 66.7%Reduced-dose: 67.5% Full dose vs. placebo: OR = 1.1 95%CI [0.7; 1.8]Reduced-dose vs. placebo: OR = 1.1 95%CI[0.7; 1.7]	CGI-I and CGI-S at week 12 (NS)	ABC-social withdrawal at week 12 (NS)	ABC-stereotypy at week 12 (NS)	ABC-irritablity and ABC-hyperactivity at week 12 (NS)	Irritability, vomiting, agitation, and anxiety	Low risk

W, Weeks; mg, milligrams; kg, kilograms; d, day; yo, years old; ABA, Applied Behaviour Analysis; ABC, Aberrant Behavior Checklist; ABC-C: ABC–Community version; ADHD, Attention Deficit/Hyperactivity Disorder; CARS: Childhood Autism Rating Scale; CATS-I: Core Autism Treatment Scale-Improvement; CCC, Children’s Communication Checklist; CGI, Clinical Global Impressions Scale: CGI-S Severity of Illness, CGI-I Global Improvement; CI, confidence intervals; GARS, Gilliam Autism Rating Scale; IQ, Intellectual Quotient; MD, mean difference; N, number; OR, odd ratio; RCT, Randomized Controlled Trial; SRS: Social Responsiveness scale; SD: Standard Deviation; VABS−2: Vineland Adaptative Behavior Scale - 2.

#### Methodology

The search retrieved 5 studies from the United States (Woodard et al., 2007; Minshawi et al., 2016; Aman et al., 2017; Gagan, 2019; Hardan et al., 2019), 3 from Iran (Ghaleiha et al., 2013; Mohammadi et al., 2013; Karahmadi et al., 2018), 1 from the UK (King et al., 2001), and 1 from Ukraine (Martsenkovsky and Martsenkovska, 2016). Six studies were unicentric (Gagan, 2019; Woodard et al., 2007; Ghaleiha et al., 2013; Karahmadi et al., 2018; Mohammadi et al., 2013; Martsenkovsky and Martsenkovska, 2016) and four were multicenter trials (King et al., 2001; Minshawi et al., 2016; Aman et al., 2017; Hardan et al., 2019), ranging from two to six different centers. The diagnosis of ASD was validated by DSM IV or 5 criteria and/or by ADI-R and/or ADOS, while some studies considered CARS (Woodard et al., 2007), GARS (Karahmadi et al., 2018), or ICD 10 (King et al., 2001) criteria.

Six studies were double-blind randomized controlled trials with intervention versus placebo. Two studies compared NMA antagonist + risperidone versus placebo plus risperidone (Ghaleiha et al., 2013; Mohammadi et al., 2013), one study was conducted using the ABAB scheme (Woodard et al., 2007), and one was a withdrawal study (Hardan et al., 2019). Eight studies had follow-up durations of ≥ 10 weeks.

The treatments evaluated were memantine in six studies (Ghaleiha et al., 2013; Martsenkovsky and Martsenkovska, 2016; Aman et al., 2017; Gagan, 2019; Karahmadi et al., 2018; Hardan et al., 2019), amantadine in two studies (King et al., 2001; Mohammadi et al., 2013), dextromethorphan in one study (Woodard et al., 2007), and d-cycloserine in one study (Minshawi et al., 2016). Risperidone was associated with amantadine in one study (Mohammadi et al., 2013) and memantine in one study (Ghaleiha et al., 2013). Two interventions were combined with behavioral therapy (Martsenkovsky and Martsenkovska, 2016; Karahmadi et al., 2018) and one with social skills training (Wink et al., 2017).

The main scales used were the Aberrant Behavior Checklist (ABC) (n = 7), Clinical Global Impression (CGI) (n = 6), Social Responsiveness Scale (SRS) (n = 5), and Children’s Communication Checklist (CCC) (n = 3). ABC, SRS, and CCC were completed by the parents and/or caregivers. CGI was rated by the investigator. No self-administered questionnaire was administered.

#### Population

Participant samples ranged from 16 to 121 and were mostly male. One study did not report sex distribution (Martsenkovsky and Martsenkovska, 2016). The patients were aged between 7 and 10 years, except for one study with participants aged 18–36 months (Martsenkovsky and Martsenkovska, 2016).

Two studies excluded patients with intellectual disability (IQ < 70) (Minshawi et al., 2016; Gagan, 2019), and two did not provide any details on IQ (Martsenkovsky and Martsenkovska, 2016; Karahmadi et al., 2018). Other studies selected participants with IQs ≥ 35. Only two studies used ADHD (Attention Deficit Hyperactivity Disorder) as an exclusion criterion (Mohammadi et al., 2013; Karahmadi et al., 2018). In other studies, this diagnosis was not sought in the inclusion criteria, the exclusion criteria, or the description of the population. Methylphenidate and other psychotropic drugs were additional exclusion criteria in some studies (Gagan, 2019; Ghaleiha et al., 2013; King et al., 2001). Only one study searched for and reported four methylphenidate users (Aman et al., 2017).

The severity of ASD according to the DSM 5 (Rosen et al., 2021) was not specified. Severity was usually assessed by CGI-Severity, but results on this scale were never reported.

### Risk of bias

**FIGURE 2:**
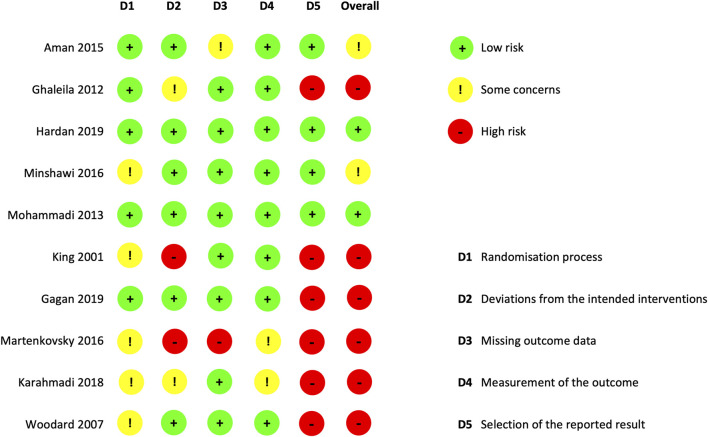
Studies risk of bias (Cochrane Risk of Bias 2 tool).

The overall quality of each study is reported in Figure 2. The risk of bias was rated as low in two studies (Mohammadi et al., 2013; Hardan et al., 2019), some concerns were reported in two studies (Minshawi et al., 2016; Aman et al., 2017), and a high risk of bias was reported in six studies (Gagan, 2019; Woodard et al., 2007; Ghaleiha et al., 2013; Karahmadi et al., 2018; King et al., 2001; Martsenkovsky and Martsenkovska, 2016). For these studies, there was a risk that the reported results had been selected *post hoc*, as they did not provide a registered protocol before the end of the study. One study (Gagan, 2019) had a registered protocol, but precision on the primary outcome was added after the end of the study. One study (Karahmadi et al., 2018) reported a subscale analysis as the primary result (ABC subscale-irritability), in contrast to the outcome described in their registered protocol (ABC total score).

### Main results reported in the included studies

#### Efficacy on main outcome

On the ten included studies, three authors did not precisely determine the main outcome of the study (Woodard et al., 2007; Martsenkovsky and Martsenkovska, 2016; Karahmadi et al., 2018).

Two studies found a significant effect of a NMDA-antagonist on irritability (rated with the ABC-irritability subscale) at week 10. In the first study, SRS-irritability at the end line was lower in the mematine + risperidone group (8.90; SD = 3.05) than in the placebo + risperidone group (12.75; SD = 3.05) (*p* ≤ 0.01) (Ghaleiha et al., 2013). In the second study, difference from baseline was higher in amantadine + risperidone group (8.60; SD = 4.65) than in placebo + risperidone group (5.35; SD = 3.95) with a mean difference of 3.2 (95%CI 0.48–6.01) (Mohammadi et al., 2013).

No significant difference in response was observed in the King et al. study, with 47% responders in the amantadine group and 37% in the placebo group {OR = 1.5 [95% CI(0.4; 5.9)]} (King et al., 2001), while Gagan (2019) reported 47% of responders in the memantine group and 19% in the placebo group.

Two studies chose the SRS-total score as the main outcome and found no significant differences between the active and placebo group. Aman et al. (2017) estimated a mean difference of −0.1 (95% CI = −7.2 to 6.6) between memantine and placebo group. Minshawi et al. (2016) estimated a mean difference of 3.61 (CI 95% = −5.95–13.17) between d-cycloserine + social skills training and placebo + social-skills training.

In the withdrawal study, no significant difference in loss of therapeutic response was observed between full-dose of memantine and placebo [OR = 1.1 (95 CI% = 0.7; 1.8)] or between reduced-dose of memantine and placebo [OR = 1.1 (95 CI% = 0.7; 1.7)] (Hardan et al., 2019).

#### Efficacy on secondary outcome

The secondary outcomes are presented in Table 1.

#### Safety and tolerability

Three studies reported serious adverse events (SAE). Aman et al. reported three SAE in the memantine group: irritability, choking, and affective disorders (Aman et al., 2017). In a study by Hardan et al. (2019), four SAE were reported in the placebo group and two in the memantine group. Suicidal thoughts were reported in one study in the placebo group (Minshawi et al., 2016).

### Meta-analysis

#### Efficacy on NMDA antagonists on autism core symptoms

**FIGURE 3:**
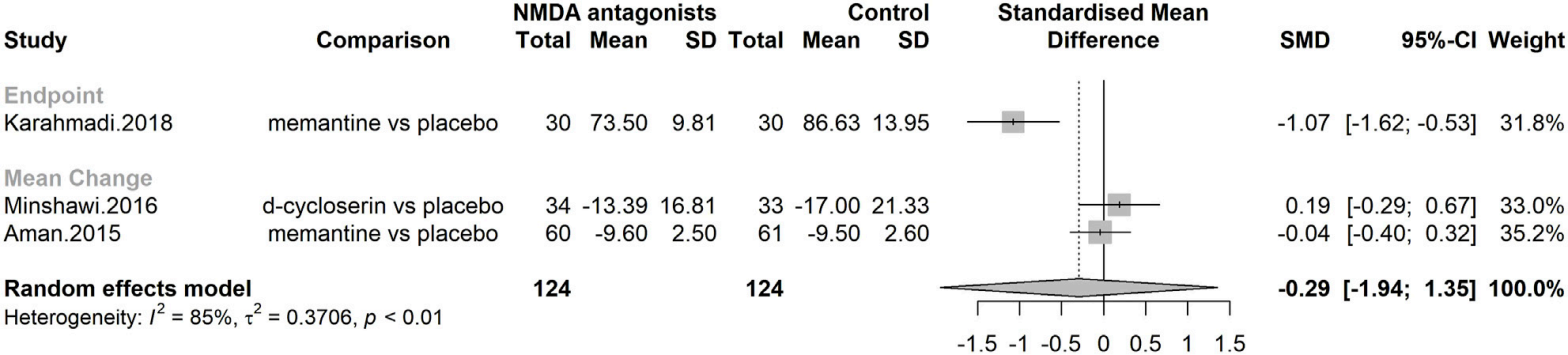
Forest plot–Efficacy on autism core symptoms.

Since many studies did not report the results of scales assessing the core symptoms of autism, three studies were included in the meta-analysis, with a total of 248 participants (Figure 3). NMDA antagonists were not superior to placebo [SMD = 0.29; CI95% (−1.94; 1.35); I^2^ = 0%].

#### Number of responders

**FIGURE 4:**
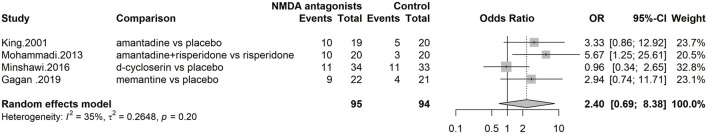
Forest plot–Number of responders.

Four studies were included in the meta-analysis of responses to NMDA antagonists, with a total of 189 participants (Figure 4). The response rate was not higher in the NMDA antagonist group than in the placebo group [OR = 2.4; CI 95% (0.69; 8.38); I^2^ = 35%].

#### Efficacy on irritability

**FIGURE 5:**
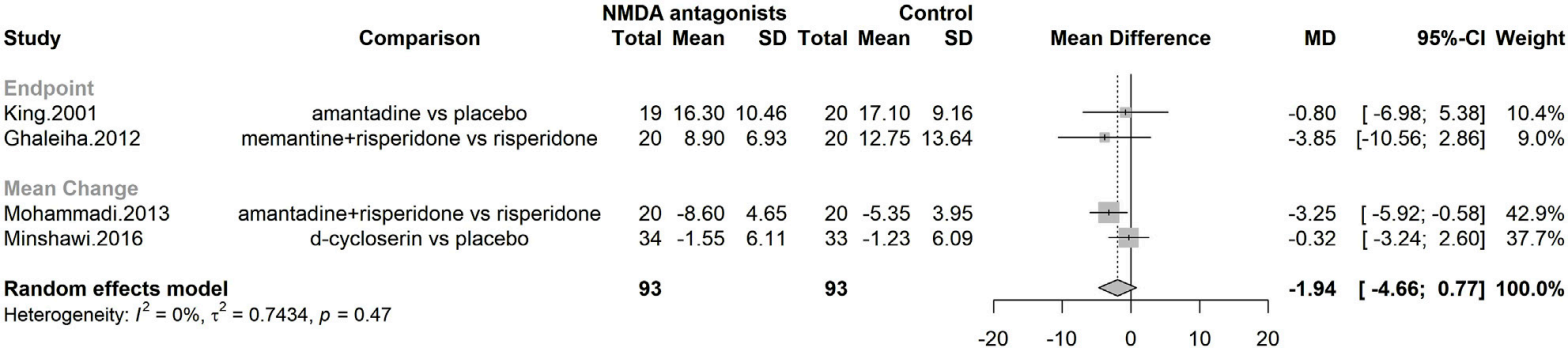
Forest plot–Efficacy on irritability.

Three studies were included in the meta-analysis, assessing the efficacy of NMDA antagonists on irritability, with a total of 186 participants (Figure 5). NMDA antagonists were not superior to placebo [MD irritability = −1.94; CI 95%(−4.66; 0.77); I^2^ = 0%].

#### At least one adverse event

**FIGURE 6:**
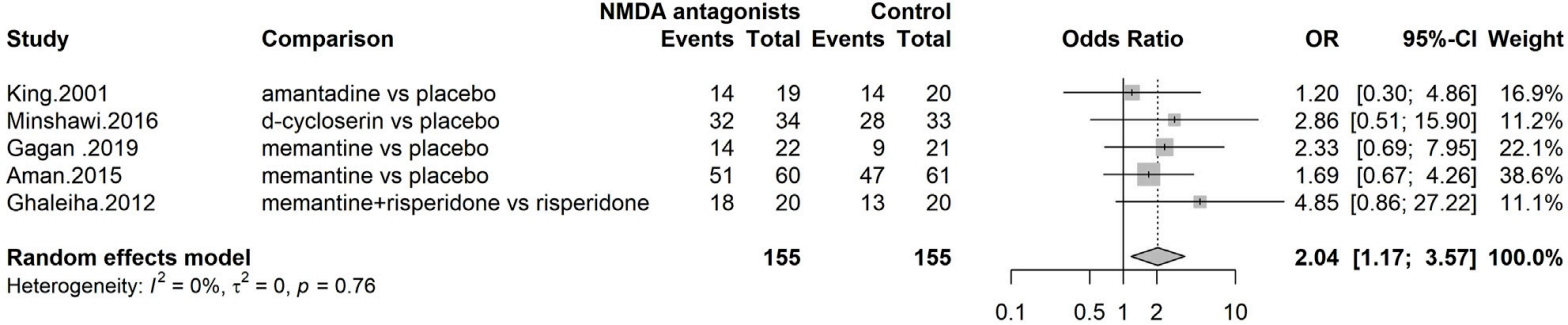
Forest plot–Number of patients with at least one adverse event.

Five studies were included in the meta-analysis of adverse events (at least one adverse event) with a total of 310 patients (Figure 6). NMDA participants had a significantly higher risk of at least one adverse event than placebo participants [OR = 2.04; CI 95% (1.17; 3.57); I^2^ = 0%].

## Discussion

The meta-analysis found no efficacy of NMDA antagonists either on the core symptoms of autism, on the overall clinical response, or on the irritability of autistic children.

These results were consistent with those reported in the literature. Two recent meta-analyses assessed the efficacy of memantine, a specific NMDA antagonist, in autism (Brignell et al., 2022; Elnaiem et al., 2022). Both studies concluded that memantine did not improve the core symptoms of autism, even if the certainty of evidence was rated as very low. No significant effects on irritability were observed.

Our review of the literature shows encouraging publications on studies with a long follow-up period (>10 weeks) using NMDA receptor antagonists as an adjuvant to other therapies (behavioral therapy, antipsychotics). These results were not synthesized in the meta-analysis because insufficient data were available.

**FIGURE 7:**
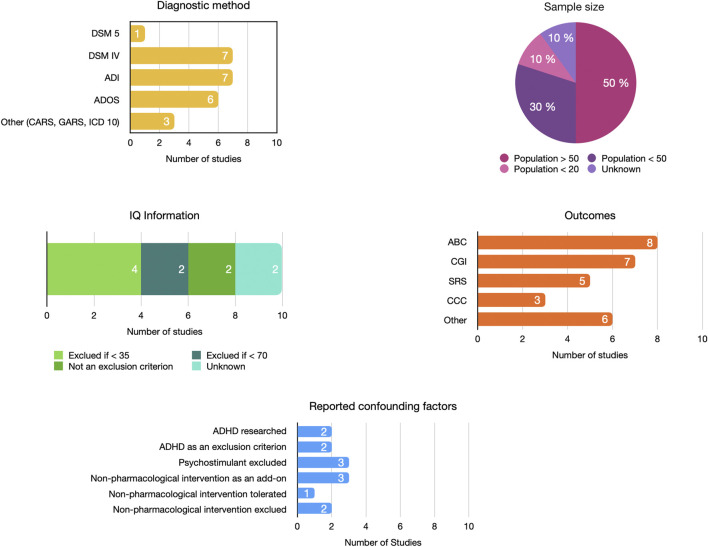
Heterogeneity in the design of included studies. ABC, Aberrant Behavior Checklist; ADHD, Attention Deficit/Hyperactivity Disorder; ADI, Autism Diagnostic Interview; ADOS, Autism Diagnostic Observation Scale; CARS, Childhood Autism Rating Scale; CCC, Children’s Communication Checklist; CGI, Clinical Global Impressions Scale; DSM, Diagnostic and Statistical Manual of Mental Disorders; GARS, Gilliam Autism Rating Scale; ICD, International Classification of Diseases; IQ, Intellectual Quotient; SRS, Social Responsiveness scale.

The variability in the results of these studies may be related to the heterogeneity of methodologies in terms of treatment, duration, population characteristics, and assessment tools (Figure 7). The methodological quality of the different studies was heterogeneous, with a high risk of bias for six studies over ten. We must point out that on several occasions, the outcomes were not ideally chosen or misinterpreted. Some studies reported subscale scores as primary outcomes, whereas the power of the study was calculated based on the total score. Other researchers have reported this observation in the ASD field (Provenzani et al., 2020). Details on the characteristics of the included population were regularly lacking; for example, the age ranges were wide, sweeping across broad levels of development and IQ. The groups did not spread out on the level of autism severity. Very little information was available on comorbidities, particularly ADHD and sleep disorders, two pathologies that could explain irritability and hyperactivity symptoms (Johnson et al., 2018; Thomas et al., 2018).

None of the included studies used self-report questionnaires completed by the children. The studies primarily relied on questionnaires filled out by parents, along with professional evaluations. This choice could be partially justified by a lack of feasibility given the high proportion of young participants and/or those with intellectual disabilities.

Concerning the safety aspects, our meta-analysis showed a higher number of patients with at least one adverse event in the NMDA antagonist group than in the control group. Some severe adverse events were reported in different studies, but none were described as “related to the drug.” These results are consistent with those of two recent meta-analyses on memantine (Brignell et al., 2022; Elnaiem et al., 2022).

### Limitations and strengths

Our study has several limitations. As the number of included studies was small, we could not proceed to the subgroup and sensibility analyses we planned. Our primary outcome (core autistic symptoms) was not described in every study, and most of the included studies focused on comorbid autism symptoms. Therefore, the meta-analyses are based on a few studies. The interventions were heterogeneous because we decided to study all NMDA receptor antagonists to understand the potential applicability of the hypothesis of a role of the NMDA system in autism.

Publication bias common to literature reviews was reduced with multiple study sources, including Clinical Trial registries, to broaden the search. Nevertheless, we could not quantitatively analyze publication bias because of the small number of included studies. Moreover, our attempts to contact different authors to retrieve missing data were unsuccessful.

Our study has several strengths. It is the first systematic review to assess the efficacy of NMDA receptor antagonists in autistic symptoms. We followed a previously published protocol on PROSPERO, and the two authors’ screening and data extractions were performed independently. The screening was updated immediately before the final analyses to retrieve potential studies for inclusion. We followed PRISMA recommendations for reporting.

### For the future

To facilitate the evaluation of therapies for autism and future study synthesis, assessment tools should be standardized, and non-validated questionnaires should not be used as the only outcome in such important studies (Provenzani et al., 2020).

It is necessary to conduct subgroup studies to evaluate treatment efficacy and tolerance according to IQ level, age, or comorbidities. In RCT concerning neurodevelopmental disorders, it is unacceptable to have an imprecise record of comorbidities (IQ, ADHD, etc.) and co-prescribed medications. There is a global need to improve study reporting to enable interpretation, comparison, and application in clinical practice.

As autism is a neurodevelopmental disorder, the literature emphasizes the importance of timing when initiating treatment to modulate brain development. Treatment effectiveness varies according to the degree of maturation and brain plasticity (Uzunova et al., 2014). Indeed, some animal models favor early treatment with NMDA antagonists to attenuate autistic symptoms (Chung et al., 2019). Due to the small number of included studies, we could not analyze the impact of different timing of treatment initiation in this review.

## Conclusion

In conclusion, the results of this meta-analysis and literature review are insufficient to confirm nor infirm the efficacy of NDMA antagonists on ASD core symptoms. The NMDA pathway remains interesting for treating behavioral symptoms associated with ASD; however, it is currently insufficiently evaluated and requires more and better-constructed studies.

The current data do not allow us to recommend the prescription of drugs for their NMDA receptor antagonist properties in the indication of ASD.

## Data Availability

The original contributions presented in the study are included in the article/Supplementary Material, further inquiries can be directed to the corresponding author.
