# Supplementary material for: NMDA antagonist agents for the treatment of symptoms in autism spectrum disorder: a systematic review and meta-analysis
Source: Front Pharmacol. 2024 Jul 23;15:1395867. doi: 10.3389/fphar.2024.1395867 (PMC11300352; doi:10.3389/fphar.2024.1395867)
Supplement: Supplementary file 1 [file Presentation1.pdf]

Supplementary file

1. PRISMA 2020 Checklist 2
2. PRISMA Checklist for abstract 5
3. Research strategies 7
a. PubMed (MEDLINE) 7
b. CENTRAL 7
c. EMBASE 8
4. References excluded 10

1. PRISMA 2020 Checklist

Section and Topic	Item #	Checklist item	Location where item is reported
**TITLE**			
Title	1	Identify the report as a systematic review.	Title
**ABSTRACT**			
Abstract	2	See the PRISMA 2020 for Abstracts checklist.	See page 5 (supp)
**INTRODUCTION**			
Rationale	3	Describe the rationale for the review in the context of existing knowledge.	Introduction part
Objectives	4	Provide an explicit statement of the objective(s) or question(s) the review addresses.	End of introduction
**METHODS**			
Eligibility criteria	5	Specify the inclusion and exclusion criteria for the review and how studies were grouped for the syntheses.	Methods: study selection (+ prosepro)
Information sources	6	Specify all databases, registers, websites, organisations, reference lists and other sources searched or consulted to identify studies. Specify the date when each source was last searched or consulted.	Methods:search strategy+supp
Search strategy	7	Present the full search strategies for all databases, registers and websites, including any filters and limits used.	supp
Selection process	8	Specify the methods used to decide whether a study met the inclusion criteria of the review, including how many reviewers screened each record and each report retrieved, whether they worked independently, and if applicable, details of automation tools used in the process.	Methods:study selection
Data collection process	9	Specify the methods used to collect data from reports, including how many reviewers collected data from each report, whether they worked independently, any processes for obtaining or confirming data from study investigators, and if applicable, details of automation tools used in the process.	Methods: data extraction
Data items	10a	List and define all outcomes for which data were sought. Specify whether all results that were compatible with each outcome domain in each study were sought (e.g. for all measures, time points, analyses), and if not, the methods used to decide which results to collect.	Methods: data extraction
	10b	List and define all other variables for which data were sought (e.g. participant and intervention characteristics, funding sources). Describe any assumptions made about any missing or unclear information.	Methods: data extraction (end)
Study risk of bias assessment	11	Specify the methods used to assess risk of bias in the included studies, including details of the tool(s) used, how many reviewers assessed each study and whether they worked independently, and if applicable, details of automation tools used in the process.	Methods: bias and quality assessement
Effect measures	12	Specify for each outcome the effect measure(s) (e.g. risk ratio, mean difference) used in the synthesis or presentation of results.	Methods: statistical analysis
Synthesis methods	13a	Describe the processes used to decide which studies were eligible for each synthesis (e.g. tabulating the study intervention characteristics and comparing against the planned groups for each synthesis (item #5)).	Methods: statistical analysis

Section and Topic	Item #	Checklist item	Location where item is reported
	13b	Describe any methods required to prepare the data for presentation or synthesis, such as handling of missing summary statistics, or data conversions.	Methods: statistical analysis
	13c	Describe any methods used to tabulate or visually display results of individual studies and syntheses.	Methods: statistical analysis
	13d	Describe any methods used to synthesize results and provide a rationale for the choice(s). If meta-analysis was performed, describe the model(s), method(s) to identify the presence and extent of statistical heterogeneity, and software package(s) used.	Methods: statistical analysis
	13e	Describe any methods used to explore possible causes of heterogeneity among study results (e.g. subgroup analysis, meta-regression).	Methods: statistical analysis
	13f	Describe any sensitivity analyses conducted to assess robustness of the synthesized results.	Methods: statistical analysis
Reporting bias assessment	14	Describe any methods used to assess risk of bias due to missing results in a synthesis (arising from reporting biases).	Methods: statistical analysis
Certainty assessment	15	Describe any methods used to assess certainty (or confidence) in the body of evidence for an outcome.	NA
**RESULTS**			
Study selection	16a	Describe the results of the search and selection process, from the number of records identified in the search to the number of studies included in the review, ideally using a flow diagram.	Results: search results+flowchart
	16b	Cite studies that might appear to meet the inclusion criteria, but which were excluded, and explain why they were excluded.	supp
Study characteristics	17	Cite each included study and present its characteristics.	Table 1 + results: Characteristics of Studies Included in the Systematic Review
Risk of bias in studies	18	Present assessments of risk of bias for each included study.	Results: risk of bias +fig2
Results of individual studies	19	For all outcomes, present, for each study: (a) summary statistics for each group (where appropriate) and (b) an effect estimate and its precision (e.g. confidence/credible interval), ideally using structured tables or plots.	Tab 1 + results: Main results reported in the included studies
	20a	For each synthesis, briefly summarise the characteristics and risk of bias among contributing studies.	Tab 1 + results: Main results

Section and Topic	Item #	Checklist item	Location where item is reported
Results of syntheses			reported in the included studies
	20b	Present results of all statistical syntheses conducted. If meta-analysis was done, present for each the summary estimate and its precision (e.g. confidence/credible interval) and measures of statistical heterogeneity. If comparing groups, describe the direction of the effect.	Fig3-6, results : Meta-analysis
	20c	Present results of all investigations of possible causes of heterogeneity among study results.	NA see deviation
	20d	Present results of all sensitivity analyses conducted to assess the robustness of the synthesized results.	NA see deviation
Reporting biases	21	Present assessments of risk of bias due to missing results (arising from reporting biases) for each synthesis assessed.	NA see deviation
Certainty of evidence	22	Present assessments of certainty (or confidence) in the body of evidence for each outcome assessed.	NA
**DISCUSSION**			
Discussion	23a	Provide a general interpretation of the results in the context of other evidence.	discussion
	23b	Discuss any limitations of the evidence included in the review.	Discussion: limitations and strengths
	23c	Discuss any limitations of the review processes used.	Discussion: limitations and strengths
	23d	Discuss implications of the results for practice, policy, and future research.	Discussion: for the future
**OTHER INFORMATION**			
Registration and protocol	24a	Provide registration information for the review, including register name and registration number, or state that the review was not registered.	Methods: protocol and registration
	24b	Indicate where the review protocol can be accessed, or state that a protocol was not prepared.	Methods: protocol and registration
	24c	Describe and explain any amendments to information provided at registration or in the protocol.	Methods: deviation from protocol
Support	25	Describe sources of financial or non-financial support for the review, and the role of the funders or sponsors in the review.	See statements at the end
Competing interests	26	Declare any competing interests of review authors.	See statements at the end

Section and Topic	Item #	Checklist item	Location where item is reported
Availability of data, code and other materials	27	Report which of the following are publicly available and where they can be found: template data collection forms; data extracted from included studies; data used for all analyses; analytic code; any other materials used in the review.	See statements at the end

2. PRISMA Checklist for abstract

Section and Topic	Item #	Checklist item	Reported (Yes/No)
**TITLE**			
Title	1	Identify the report as a systematic review.	yes
**BACKGROUND**			
Objectives	2	Provide an explicit statement of the main objective(s) or question(s) the review addresses.	yes
**METHODS**			
Eligibility criteria	3	Specify the inclusion and exclusion criteria for the review.	yes
Information sources	4	Specify the information sources (e.g. databases, registers) used to identify studies and the date when each was last searched.	yes
Risk of bias	5	Specify the methods used to assess risk of bias in the included studies.	yes
Synthesis of results	6	Specify the methods used to present and synthesise results.	yes
**RESULTS**			
Included studies	7	Give the total number of included studies and participants and summarise relevant characteristics of studies.	yes
Synthesis of results	8	Present results for main outcomes, preferably indicating the number of included studies and participants for each. If meta-analysis was done, report the summary estimate and confidence/credible interval. If comparing groups, indicate the direction of the effect (i.e. which group is favoured).	yes
**DISCUSSION**			
Limitations of evidence	9	Provide a brief summary of the limitations of the evidence included in the review (e.g. study risk of bias, inconsistency and imprecision).	yes
Interpretation	10	Provide a general interpretation of the results and important implications.	yes
**OTHER**			
Funding	11	Specify the primary source of funding for the review.	no

Section and Topic	Item #	Checklist item	Reported (Yes/No)
Registration	12	Provide the register name and registration number.	yes

3. Research strategies

a. PubMed (MEDLINE)

#1	"autism spectrum disorder"[MeSH Terms]
#2	"child development disorders, pervasive"[MeSH Terms]
#3	("asd"[Title/Abstract]) OR "autism" OR "autistic disorder" OR ("autistic"[Title/Abstract])
#4	"pervasive developmental disorder"
#5	“PDD”[Title/Abstract]
#6	#1 OR #2 OR #3 OR #4 OR #5 OR #6
#7	("amantadine"[MeSH Terms]) OR "amantadine"[Title/Abstract]
#8	("phencyclidine"[MeSH Terms]) OR "phencyclidine"[Title/Abstract]
#9	("ethanol"[MeSH Terms]) OR "ethanol"[Title/Abstract]
#10	("procaine"[MeSH Terms]) OR "procaine"[Title/Abstract]
#11	("dextromethorphan"[MeSH Terms]) OR "dextromethorphan"[Title/Abstract]
#12	("ketamine"[MeSH Terms]) OR "ketamine"[Title/Abstract]
#13	"profenamine"[Title/Abstract]
#14	("methadone"[MeSH Terms]) OR "methadone"[MeSH Terms]
#15	("felbamate"[MeSH Terms]) OR "felbamate"[Title/Abstract]
#16	"ifenprodil"[Title/Abstract]
#17	("agmatine"[MeSH Terms]) OR "agmatine"[Title/Abstract]
#18	"tenocyclidine"[Title/Abstract]
#19	("orphenadrine"[Title/Abstract]) OR "orphenadrine"[MeSH Terms]
#20	("acamprosate"[MeSH Terms]) OR "acamprosate"[Title/Abstract]
#21	("memantine"[MeSH Terms]) OR "memantine"[Title/Abstract]
#22	#7 OR #8 OR #9 OR #10 OR #11 OR #12 OR #13 OR #14 OR #15 OR #16 OR #17 OR #18 OR #19 OR #20 OR #21
#23	#6 AND #22

b. CENTRAL

#1 MeSH descriptor: [Child Development Disorders, Pervasive] explode all trees
#2 autism

#3 "pervasive developmental disorder"
#4 autistic
#5 amantadine
#6 memantine
#7 acamprosate
#8 orphenadrine
#9 tenocyclidine
#10 agmatine
#11 ifenprodil
#12 felbamate
#13 methadone
#14 profenamine
#15 ketamine
#16 dextromethorphan
#17 procaine
#18 ethanol
#19 phencyclidine
#20 (#1 OR #2 OR #3 OR #4)
#21 (#5 OR #6 OR #7 OR #8 OR #9 OR #10 OR #11 OR #12 OR #13 OR #14 OR #15 OR #16 OR #17 OR #18 OR #19)
#22 #20 AND #21 in Trials

c. EMBASE

('autism spectrum disorder':ti,ab,kw OR 'child development disorders, pervasive':ti,ab,kw OR 'asd':ti,ab,kw OR 'autism':ti,ab,kw OR 'autistic disorder':ti,ab,kw OR 'autistic':ti,ab,kw OR 'pervasive developmental disorder':ti,ab,kw OR 'pdd':ti,ab,kw) AND (amantadine:ti,ab,kw OR 'phencyclidine':ti,ab,kw OR 'ethanol':ti,ab,kw OR 'procaine':ti,ab,kw OR 'dextromethorphan':ti,ab,kw OR 'ketamine':ti,ab,kw OR 'profenamine':ti,ab,kw OR 'methadone':ti,ab,kw OR 'felbamate':ti,ab,kw OR 'ifenprodil':ti,ab,kw OR 'agmatine':ti,ab,kw OR 'tenocyclidine':ti,ab,kw OR 'orphenadrine':ti,ab,kw OR 'acamprosate':ti,ab,kw OR 'memantine':ti,ab,kw) AND [humans]/lim AND [embase]/lim NOT review:it

d. EBSCOhost: PsycINFO and ERIC

("autism spectrum disorder" OR "child development disorders, pervasive" OR "asd" OR "autism" OR "autistic disorder" OR "autistic" OR "pervasive developmental disorder" OR “PDD”) AND ("amantadine" OR "phencyclidine" OR "ethanol" OR "procaine" OR “dextromethorphan" OR "ketamine" OR "profenamine" OR "methadone" OR "felbamate" OR "ifenprodil" OR "agmatine" OR "tenocyclidine" OR "orphenadrine" OR "acamprosate" OR OR "memantine")

4. References excluded.

Title	Study	Notes
Brief Report: Intranasal Ketamine in Adolescents and Young Adults with Autism Spectrum Disorder-Initial Results of a Randomized, Controlled, Crossover, Pilot Study	Wink 2020	Exclusion reason: Wrong patient population; 14 to 29 years (M = 19.48, SD = 3.83);
5.14 RESULTS OF A DOUBLE-BLIND, PLACEBO-CONTROLLED CROSSOVER STUDY OF INTRANASAL KETAMINE IN ADOLESCENTS AND YOUNG ADULTS WITH ASD	Wink 2019	Exclusion reason: Wrong patient population Duplicate population; conference paper, same as Wink 2020;
Results of a Double-Blind, Placebo-Controlled Crossover Study of Intranasal Ketamine in Adolescents and Young Adults With Autism Spectrum Disorder	Wink 2019	Exclusion reason: Adult population; same as wink 2020
Comparing Efficacy and Side Effects of Memantine vs. Risperidone in the Treatment of Autistic Disorder	Nikvarz 2017	Exclusion reason: Wrong study design;
Glutamate antagonists seem to be slightly effective in psychopharmacologic treatment of autism	Niederhofer 2007	Exclusion reason: Wrong study design;
Study of Acamprosate in Autism	NCT01813318	Exclusion reason: No result available → report not retrieved
Memantine for the Treatment of Social Deficits in Youth With Disorders of Impaired Social Interactions	NCT03553875	Exclusion reason: Study still recruiting;
Intranasal Ketamine With Dexmedetomidine for the Treatment of Children With Autism Spectrum Disorder	NCT03434366	Exclusion reason: Wrong intervention;
Study of Intranasal Ketamine for Social Impairment in Autism Spectrum Disorder	NCT02611921	Exclusion reason: Wrong patient population, Duplicate report; same as #Wink 2020
Safety Study of Memantine in Pediatric Patients With Autism, Asperger's Disorder or Pervasive Developmental Disorder Not Otherwise Specified (PDD-NOS)	NCT01592773	Exclusion reason : wrong study design (open-lapel)
Memantine for the treatment of autism spectrum disorder: overview of the phase II clinical development program	Katz 2014	Exclusion reason: Wrong article type, Review of 4 protocols;

Comparing efficacy and side effects of Memantine and Risperidone in treating autistic patients	Irct201204037202N 2012	Exclusion reason: Wrong setting;
Glutamatergic medication in the treatment of obsessive compulsive disorder (OCD) and autism spectrum disorder (ASD) - study protocol for a randomised controlled trial	Häge 2016	Exclusion reason: study stopped due to insufficient inclusions
Does the Clinical Benefit of Ketamine Treatment Offer Any Clues to Autism Spectrum Disorder Etiology?	Fluegge 2016	Exclusion reason: Wrong study design;
Glutamatergic medication in the treatment of Obsessive Compulsive Disorder (OCD) and Autism Spectrum Disorder (ASD)	Euctr 2014	Exclusion reason: Same as Häge A, study not completed
Brief Report: Pilot Single-Blind Placebo Lead-In Study of Acamprosate in Youth with Autistic Disorder	Erickson 2014	Exclusion reason: Wrong study design;
Memantine for disruptive behavior in autistic disorder	Erickson 2006	Exclusion reason: Wrong study design; Adulte case report and no RCT;
Memantine for comorbid obsessive-compulsive disorder and Asperger disorder suggests a link in glutamatergic dysregulation	Bernhardt 2011	Exclusion reason: Wrong study design;
Treating autism with dextromethorphan	Brown University Child & Adolescent Psychopharmacology Update 2015	Exclusion reason: Wrong study design;
A Multisite Double-blind Placebo-controlled Trial of Memantine Versus Placebo in Children With Autism (MEM)	Anagnostou 2017, NCT01372449	Exclusion reason: Wrong outcome (cognitive test only)
